# Tn-sequencing of *Mycoplasma hyopneumoniae* and *Mycoplasma hyorhinis* mutant libraries reveals non-essential genes of porcine mycoplasmas differing in pathogenicity

**DOI:** 10.1186/s13567-019-0674-7

**Published:** 2019-07-19

**Authors:** Bettina S. Trueeb, Simona Gerber, Dominiek Maes, Walid H. Gharib, Peter Kuhnert

**Affiliations:** 1 Institute of Veterinary Bacteriology, Vetsuisse Faculty, University of Bern, Bern, Switzerland; 2 Unit Porcine Health Management, Department of Reproduction, Obstetrics and Herd Health, Faculty of Veterinary Medicine, Ghent University, Ghent, Belgium; 3 Interfaculty Bioinformatics Unit and Swiss, Institute of Bioinformatics, University of Bern, Bern, Switzerland; 4 Graduate School for Cellular and Biomedical Sciences, University of Bern, Bern, Switzerland

## Abstract

*Mycoplasma hyopneumoniae* and *Mycoplasma hyorhinis* are two phylogenetically related species colonizing the respiratory tract of pigs but differing in pathogenicity, the basis of which is not well resolved. We hypothesize that genes belonging to the species-specific portion of the genome and being non-essential during ideal laboratory growth conditions encode possible virulent determinants and are the driver of interspecies differences. To investigate this, transposon mutant libraries were generated for both species and a transposon sequencing (Tn-seq) method for mycoplasmas was established to identify non-essential genes. Tn-seq datasets combined with bidirectional Blastp analysis revealed that 101 out of a total 678 coding sequences (CDS) are species-specific and non-essential CDS of *M. hyopneumoniae* strain F7.2C, while 96 out of a total 751 CDS are species-specific and non-essential CDS in the *M. hyorhinis* strain JF5820. Among these species-specific and non-essential CDS were genes involved in metabolic pathways. In particular, the myo-inositol and the sialic acid pathways were found to be non-essential and therefore could be considered important to the specific pathogenicity of *M.* *hyopneumoniae* and *M. hyorhinis*, respectively. Such pathways could enable the use of an alternative energy source providing an advantage in their specific niche and might be interesting targets to knock out in order to generate attenuated live vaccines.

**Electronic supplementary material:**

The online version of this article (10.1186/s13567-019-0674-7) contains supplementary material, which is available to authorized users.

## Introduction

Mycoplasmas form a genus of bacteria that has undergone reductive evolution from Gram-positive bacteria to wall-less cells with a small genome of high AT content. They are highly host-adapted and often host-dependent [1]. As a result of their parasitic lifestyle, mycoplasmas have lost a considerable part of their genome, retaining genes that are mostly essential for growth and replication [2].

*Mycoplasma hyopneumoniae* and *M.* *hyorhinis* are two phylogenetically related species found in the respiratory tract of pigs, but differing in pathogenicity [3, 4]. *M. hyopneumoniae* is the etiological agent of enzootic pneumonia (EP), a chronic insidious bronchopneumonia characterized by non-productive cough [5]. In contrast, *M.* *hyorhinis* is mainly found as a commensal in the respiratory tract of pigs without causing disease [6], but can also be isolated from clinical cases of arthritis, polyserositis, eustachitis and otitis [7]. The chronic nature of these disease conditions suggests difficulties of the host immune system to rapidly clear the mycoplasma infection. In consequence, both porcine *Mycoplasma* species are the cause of major economic losses to swine producers worldwide and no fully protective vaccines against either *Mycoplasma* species are available to date. The overall goal of this study was to identify potential candidate genes for the generation of an attenuated live vaccine using an innovative vaccine approach. To select relevant candidate genes of *M. hyopneumoniae* or *M. hyorhinis* and better explain their difference in pathogenicity, we aimed to identify the species-specific and non-essential genes of both species. We consider the species-specific portion of the genome of these phylogenetically related species being responsible for their difference in pathogenicity. Furthermore, we consider that virulence associated genes, e.g. for adhesion, invasion, toxin production etc. are non-essential for bacteria grown under ideal laboratory conditions. Hence, the species-specific set of genes for both *Mycoplasma* species was identified by bidirectional BLASTp analysis of their protein files and the non-essential genes of *M.* *hyopneumoniae* strain F7.2C and of *M. hyorhinis* strain JF5820 were investigated by sequencing corresponding transposon mutant libraries.

## Materials and methods

### Mycoplasma strains and cultivation

The highly virulent *M. hyopneumoniae* strain F7.2C was received from the laboratory of Bacteriology, Faculty of Veterinary Medicine, Ghent University, Belgium. It was isolated in Belgium in 2000 at slaughter from a pig with typical EP lesions [8]. The *M. hyorhinis* strain JF5820 (Ue1435_15) was isolated in Switzerland in 2015 from the lung of a pig under suspicion but negative for EP. Both strains were stored at −80 °C until usage. Strains were grown separately in liquid medium (Mycoplasma Experience, Bletchingley, Great Britain) in a static incubator at 37 °C until medium color change from red to orange occurred. Alternatively colonies were grown on solid medium agar plates (Mycoplasma Experience) incubated at 37 °C and 5% CO_2_. For the selection of transposon mutants tetracycline hydrochloride (Sigma-Aldrich Ltd, Gillingham, Great Britain; 1 mg/mL stock in 70% of ethanol) was added to liquid medium to achieve a final concentration of 0.3 µg/mL for *M. hyopneumoniae* and 3 µg/mL for *M. hyorhinis*. The same concentrations were used for agar plates.

### Genome sequencing and annotation

Both mycoplasma strains were cultured in 200 mL liquid medium at 37 °C until color change occurred. Genomic DNA was then extracted using the peqGOLD Bacterial DNA Kit (VWR International GmbH, Vienna, Austria) and sent for PacBio sequencing to the Lausanne Genomic Technologies Facility, located at the Center for Integrative Genomics of the University of Lausanne, Switzerland.

For *M. hyopneumoniae* strain F7.2C a total of 105 900 reads was obtained with a mean read length of 13 775 bp, covering a total of 1 458 810 878 bp. The mean coverage was 1180×. The final assembled genome consisted of a single contig of 925 330 bp and was then circularized with to Amos to a chromosome of 894 983 bp. For *M. hyorhinis* strain JF5820 a total of 105 433 reads was obtained with a mean read length of 18 496 bp, covering a total of 1 950 150 540 bp. The mean coverage was 1289×. The final assembled genome consisted of a single contig of 852 181 bp and was then circularized with to Amos to a chromosome of 840 423 bp. Both genomes were automatically annotated using a Prokka software program code 4. For the annotation of the genome of *M.* *hyopneumoniae* strain F7.2C the genome of strain J (acc. no. NC_007295) was used as reference and for the one of *M. hyorhinis* strain JF5820 the genome of strain SK76 (acc. no. NC_019552) was used as a reference. Genome sequences are deposited under accession number CP034597 and CP035041, respectively.

### Generation of plasmid pMT95res

**Figure 1:**
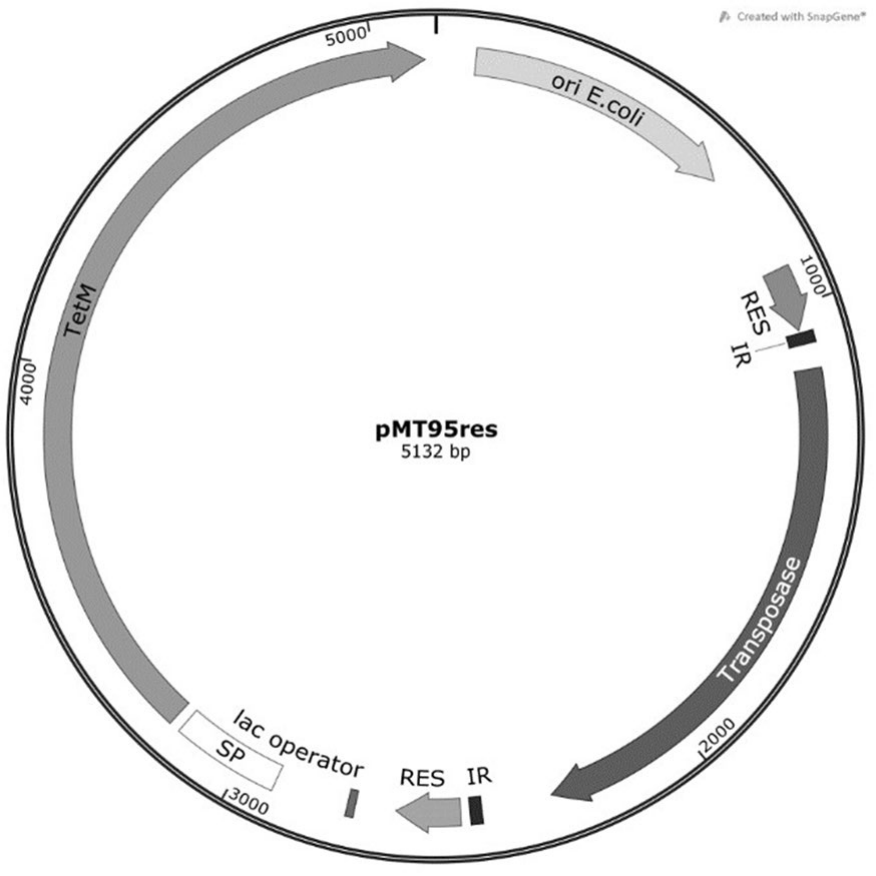
**Graphical representation of the plasmid pMT95res.** The plasmid contains an *oriC* for replication in *E. coli* and a *tetM* gene allowing for selection of transposon mutants with tetracycline, the *res* sequences would allow for generating unmarked mutants by removing *oriC* and *tetM.*

The *aacA*-*aphD* resistance cassette of plasmid pMT85/2res (kindly provided by Dr Pascal Sirand-Pugnet, Université de Bordeaux, France) [9], which is situated between two resolvase (res) recognition sites was replaced by a tetracycline resistance cassette *tetM*. We termed the new plasmid pMT95res (Figure 1). This was done to later have the possibility to remove the tetracycline resistance cassette from the transposon mutants. For this the backbone of pMT85/2res was multiplied with primers designed to amplify the plasmid without the *aacA*-*aphD* gene (GATCTACGAAGGCATGACCAAAAATC and GTTATCCGCACAATTCACAC) using Phusion^®^ High-Fidelity DNA Polymerase (Thermo Fisher Scientific, Reinach, Switzerland). In parallel, the *tetM* gene was amplified from pMT85 [10] with homology or with adding flanking homology of 20 to the backbone on both ends using corresponding primers (ATATTGTGTGGAATTGTGAG and GATTTTGGTCATGCCTTCGTAGATCTTTATATAACAACTTAAATTACAC). The amplification templates (pMT85/2res and pMT85) were then digested with *Dpn*I. Finally, the backbone and *tetM* fragments were mixed in a 1:5 ratio and transformed into competent *E. coli* DH5α cells. The natural recombination machinery of *E. coli* DH5α cells assembled pMT95res. Afterwards, pMT95res was sequenced to confirm the correct insertion of *tetM*. Plasmid pMT95res is therefore a derivate of pMT85 and contains the mini transposon Tn4001 [11].

### Generating transposon mutant libraries

The method for transformation of both*, M. hyopneumoniae* and *M. hyorhinis*, was modified from Dybvig and Alderete [12]. *M. hyopneumoniae* strain F7.2C and *M. hyorhinis* strain JF5820 were cultured in 6 mL liquid medium in 15 mL Falcon tubes and grown at 37 °C until color change. Cells were harvested by centrifugation at 3500 × *g* for 20 min at 4 °C. The supernatants were discarded and the pellets washed three times with 1 mL ice-cold PBS (Sigma-Aldrich Ltd, Gillingham, Great Britain) in Eppendorf tubes. In between the washes, the cells were centrifuged at 10 000 × *g* for 10 min at 4 °C. The pellets were finally re-suspended in 100 μL ice-cold 0.1 M CaCl_2_ (Sigma-Aldrich Ltd) and 0.02 mM beta-mercaptoethanol (Sigma-Aldrich Ltd). After incubation for 20 min on ice, mycoplasmas were added to the transformation mixture containing 8 µg plasmid pMT95res and 15 µg tRNA (Sigma-Aldrich Ltd). Transformation reactions were incubated on ice for 10 min with gentle flicking every 2 min. Afterwards they were transferred to 50 mL Falcon tubes containing 1 mL 50% Polyethylene glycol 8000 (Sigma-Aldrich Ltd), gently vortexed and left at room temperature for exactly 1 min. Five millilitre fresh medium was added and the transformation reactions of *M. hyopneumoniae* were incubated for 3 h, while those of *M. hyorhinis* were incubated for 1.5 h at 37 °C. Thereafter, mycoplasmas were harvested by centrifugation at 3500 × *g* for 15 min at 4 °C and the pellets re-suspended in 1 mL fresh medium. After an incubation time of 10 min at 37 °C, 100 µL of the transformation reactions were plated on agar plates containing the corresponding tetracycline concentration. Plates were incubated at 37 °C, 5% CO_2_ allowing growth of tetracycline resistant transposon mutants. After 10 days of incubation for *M.* *hyopneumoniae* and 5 days for *M.* *hyorhinis*, individual tetracycline resistant colonies were picked using sterile pipette tips and grown in 96-well plates with 200 μL liquid medium containing the corresponding tetracycline concentration. Following this protocol, approximately fifty 96-well plates were filled with transposon mutants of each *Mycoplasma* species; these 96-well plates make up the transposon mutant libraries and were stored at −80 °C until further usage.

### Genomic DNA isolation from transposon mutant libraries

The transposon mutant libraries of *M. hyopneumoniae* and *M. hyorhinis* were thawed and replicated on new 96-well plates containing 200 µL medium supplemented with either 0.3 µg/mL tetracycline for *M.* *hyopneumoniae* plates or 3 µg/mL tetracycline for *M. hyorhinis* plates. Plates were inoculated with the transposon mutant libraries cultured at 37 °C in wet chambers for 8 days (*M. hyopneumoniae*) or 5 days (*M.* *hyorhinis*).

Five transposon mutant plates at a time were harvested and pooled into 50 mL Falcon tubes. The genomic DNA was isolated using the DNeasy Blood & Tissue Kit (QIAGEN GmbH, Hilden, Germany) following the protocol for pretreatment of Gram-positive bacteria except that the step for bacterial cell wall lysis was skipped and the extracted DNA was eluted in water.

### Localization of transposon insertion sites by Tn-seq

**Figure 2:**
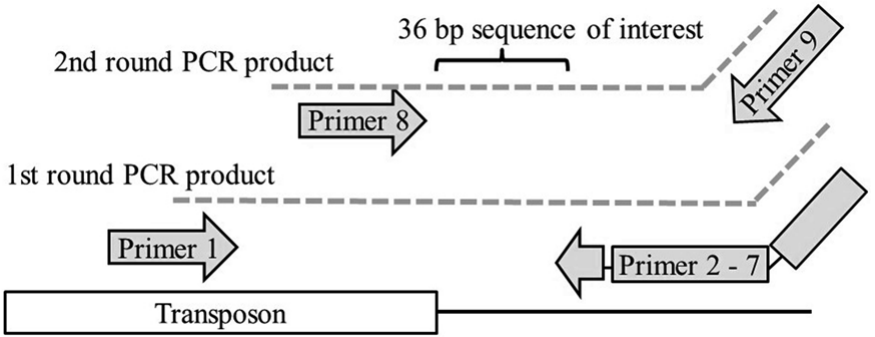
**Tn-sequencing schematic representation.** A two round PCR followed by Illumina sequencing was done. The first round is done with a Tn-specific and arbitrary outside primers, the second round with nested primers. This PCR product is then Illumina-sequenced resulting in 36 bp target gene sequence.

In order to amplify the flanking sequences of the transposon insertion site, two subsequent PCRs were performed on the pooled DNA of the mutant library. The method was adapted from Christen et al. [13]. One transposon specific primer and six semi-arbitrary primers were designed for the first round of PCR. The semi-arbitrary primers are composed of 3′ pentanucleotides flanked by 10 high-fidelity wobble bases with 72% AT skew and tail (Table 1). The first round PCR was performed in a total volume of 60 µL with 6 µL genomic DNA as template. The nested second round PCR was performed in a total volume of 30 µL with 2 µL of the PCR products from the first round (Table 1). The PCR mixtures were prepared with 1× FIREPol^®^ Master Mix Ready to load (Solis Biodyne, Tartu, Estonia) containing 2.5 mM MgCl_2_ and 0.4 µM of each primer. An extra 2.5 mM MgCl_2_ was added to the first round of PCR (Table 2 and Figure 2). Thereafter, the PCR products were purified with the High Pure PCR Product Purification Kit (Roche Diagnostics GmbH, Mannheim, Germany) and sent to Microsynth (Balgach, Switzerland) for barcoding of samples and Illumina sequencing. After removing primer and transposon sequence, the reads contained 36 bp of genomic sequence flanking the transposon.

**Table 1:** **Primer list for 1st round PCR and 2nd round PCR for Tn-seq**

No	Primer	Sequence
1	NGS_1st_Seq6	GACTTGAGCGTCGATTTTTGTG
2	NGS_1st_PCR_rev_1	GTCTCGTGGGCTCGGAGATGTGTATAAGAGACAG-NNNNNNNNNN-TGATT
3	NGS_1st_PCR_rev_2	GTCTCGTGGGCTCGGAGATGTGTATAAGAGACAG-NNNNNNNNNN-TTGAT
4	NGS_1st_PCR_rev_3	GTCTCGTGGGCTCGGAGATGTGTATAAGAGACAG-NNNNNNNNNN-TTTAG
5	NGS_1st_PCR_rev_4	GTCTCGTGGGCTCGGAGATGTGTATAAGAGACAG-NNNNNNNNNN-CAGG
6	NGS_1st_PCR_rev_5	GTCTCGTGGGCTCGGAGATGTGTATAAGAGACAG-NNNNNNNNNN-TTCCC
7	NGS_1st_PCR_rev_6	GTCTCGTGGGCTCGGAGATGTGTATAAGAGACAG-NNNNNNNNNN-TCAGC
8	Seq7_NGS_tail_F	TCGTCGGCAGCGTCAGATGTGTATAAGAGACAG-GTTGCGGTACCCTTTTACAC
9	NGS_tail_R	GTCTCGTGGGCTCGGAGATGTGTATAAGAGACAG

**Table 2:** **Two round PCR program**

Step	°C	Time	Go to
1^st^ round PCR			
1	95	4 min	
2	95	30 s	
4	42	30 s and slope −2 °C per cycle	
5	72	1.5 min	Step 2, 6 times
6	95	30 s	
7	55.8	30 s	
8	72	1.5 min	Step 7, 20 times
9	72	5 min	
2^nd^ round PCR			
1	95	4 min	
2	95	30 s	
3	59	30 s	
4	72	1 min	Step 2, 25 times
5	72	5 min

### Identification of species-specific CDS of either the *M. hyopneumoniae* strain F7.2C or *M. hyorhinis* strain JF5820

Bidirectional Blastp was performed on *.faa files of the whole genome sequence of the two species [14]. CDS of protein sequences with a Blastp resulting in E-values smaller than e^−5^ in both directions were treated as shared CDS among *M. hyorhinis* and *M. hyopneumoniae*, whereas all CDS not matching the criteria were classified as species-specific, either for *M. hyorhinis* or *M. hyopneumoniae*. Species-specificity refers to CDS only found in one or the other of the two, *M.* *hyopneumoniae* strain F7.2C or *M. hyorhinis* strain JF5820. Possible strain specific differences in CDS within species were not considered in this study.

### Essentiality analysis

Gene essentiality computations were performed using custom scripts located at GitHub [15] and originally developed by Turner et al. [16]. Raw sequencing data for both *M. hyopneumoniae* and *M. hyorhinis* transposon mutants were trimmed/filtered according to the presence of the primer sequences used for amplification and the transposon tag and aligned against their respective reference genomes using bowtie2 [17]. A Monte Carlo simulation method was used to simulate insertion sites across the genome and generate an expected pseudo dataset (repeated 500 times). The Monte Carlo simulations are considered as the expected values for non-essential genes. The R package EdgeR [18] was used to compare the differences of abundance between the real (mutant) and pseudo data sets, this package uses a negative binomial distribution to assign log fold changes. A gene was assigned as essential in case of a negative log fold change, in other words, the number of reads over this gene in the real data were less abundant than the simulated pseudo-dataset counts. Log fold changes larger than −3.69 for the Tn-seq dataset of *M. hyopneumoniae* and larger than −3.88 for the Tn-seq dataset of *M. hyorhinis* were assigned as non-essential CDS. For this, we refer to CDS being non-essential as CDS that are individually dispensable for the mycoplasma grown under ideal laboratory conditions. The essentiality classification based on Tn-seq data sets remains on a putative level, since insertion of CDS by transposons were not analyzed for functional disruption of the gene.

## Results

### Genome sequences of *M. hyopneumoniae* strain F7.2C and of *M. hyorhinis* strain JF5820

As a basis for essentiality analysis using Tn-seq of transposon mutant libraries, the genomes of the two strains were sequenced. This resulted in a 894 983 bp genome for the *M. hyopneumoniae* strain F7.2C with an average GC-content of 27.7%, 678 predicted protein coding genes as well as genes for 32 tRNA and 3 rRNA. The genome of strain F7.2C harbours six operons of the P97/P102 adhesin family. Moreover, six IS1634-like/ISMhp1 family transposase CDS and four IS3 family transposase CDS were found in the genome. The genome of *M. hyorhinis* strain JF5820 consisted of 840 423 bp with an average GC-content of 21.9%. The genome contained 751 predicted protein coding genes as well as genes for 30 tRNA, 3 rRNA and 1 tmRNA. *M. hyorhinis* strain JF5820 contained genes for a variable lipoprotein (Vlp) system that is composed of the *vlpC*-*vlpB*-*vlpA*-*vlpG*-mobile element-IS3 and *vlpC*-*vlpB*-IS3-*vlpA*-*vlpE*-mobile element-IS3-*vlpF*-*vlpE*-*vlpD*. In total, 39 IS1634-like/ISMhp1 family transposases were found in the genome sequence of *M. hyorhinis* strain.

### Localization of transposon insertion sites by Tn-seq of transposon mutant libraries

Tn-seq was used to reveal 5614 unique transposon insertions in the mutant library of the *M.* *hyopneumoniae* strain F7.2C and 4756 unique transposon insertions in the mutant library of the *M.* *hyorhinis* strain JF5820. The transposon insertion frequency was calculated by dividing the genome size by the number of unique insertions. It resulted in a hypothetical transposon insertion every 159 bp for the *M. hyopneumoniae* strain F7.2C and every 183 bp for the *M. hyorhinis* strain JF5820. Bidirectional BLASTp analysis of the protein files was performed in order to define the species-specific portion of the genome. For *M. hyopneumoniae* strain F7.2C, out of 678 CDS, 140 were identified as species-specific of which 101 CDS were non-essential under ideal growth conditions. Out of these 101 non-essential CDS, 75 were annotated as hypothetical proteins (Additional file 1). In parallel, 187 species-specific CDS for *M. hyorhinis* strain JF5820 were identified, of which 96 were classified as non-essential and 59 CDS out of these being annotated as hypothetical proteins (Additional file 2).

### CDS classified as species-specific and non-essential for *M. hyopneumoniae*

CDS being species-specific and non-essential to *M. hyopneumoniae* are listed in Additional file 1. Six genes of the myo-inositol pathway, namely *iolA, iolC, iolB, iolD, iolE and iolX* (locus tag EHI52_02600-02620; _02640; _02650; _02690) are encoded in the genome of *M.* *hyopneumoniae* and all of them were classified as non-essential. Similarly, the pathway for the uptake and catabolism of GlcNAc with *nagA* and *crr* (locus tag EHI52_06070; _06490), were non-essential. Furthermore, two serine protease genes (locus tag EHI52_04340; _06040) encoded in the genome of *M.* *hyopneumoniae* were non-essential. Two CDS encoding transport systems are species-specific and non-essential for *M. hyopneumoniae*, namely one MFS transporter (locus tag EHI52_05310) out of three and the PTS galacitol transporter subunit IIB (locus tag EHI52_05900). DNA restriction modification systems, which are known to protect mycoplasmas from invading foreign DNA are also part of the species-specific and non-essential gene pool, namely Type-2 restriction enzyme *Bsu*MI, component *ydiS* (locus tag EHI52_ 07120) and a SAM-dependent DNA methyltransferase CDS (locus tag EHI52_03280). Moreover, six transposable elements of the IS1634-like/ISMhp1 family transposase (locus tag EHI52_01210; _02370; _03590; _04360; _06050; _06970) are all classified as non-essential and only found in *M. hyopneumoniae* but not in *M.* *hyorhinis*. Next to these six transposable elements, both species encode multiple IS3 family transposase. *M. hyopneumoniae* encodes six operons of the p97/p102 family, the gene products of which have been shown to be involved in adhesion, extracellular matrix binding and surface variation. Three paralogs of p102 including the paralog annotated as p116 (locus tag EHI52_03050; _03780; _06980) were classified as species-specific and non-essential. The gene *glpK* (locus tag EHI52_03900) involved in the pathway for interconversion of glycerol into H_2_0_2_ is classified non-essential.

### CDS classified as species-specific and non-essential to *M. hyorhinis*

CDS being species-specific and non-essential to *M. hyorhinis* are listed in Additional file 2. These include CDS involved in the sialic acid pathway. In particular, genes for an exo-alpha-sialidase_2 (locus tag EIH16_05270), sialic acid transporter_2 (locus tag EIH16_04990), N-acetylneuraminate lyase (*nanA_1* and *nanA_2*, locus tag EIH16_05010; _05020), N-acetylmannosamine kinase (*nanK_2*, locus tag EIH16_05040), and N-acetylmannosamine-6-phosphate 2-epimerase (*nanE_1*, locus tag EIH16_05040) were found to be non-essential to *M. hyorhinis*. Several CDS of the sialic acid scavenging and degradation pathway are found twice in *M. hyorhinis*. The genes for α-amylase (locus tag EIH16_05340) and sucrase-isomaltase (locus tag EIH16_01870), both being involved in glycogen catabolism, are encoded in *M. hyorhinis* and are non-essential. In addition, a glycosyl transferase (locus tag EIH16_0870), which links carbohydrate residues to other carbohydrates, lipids, nucleic acids and proteins, is non-essential. Further, an acid and a serine/threonine-protein phosphatase (locus tag EIH16_06950) are non-essential. The species-specific genes for *vlpA*, *vlpC*, *vlpE*, *vlpF*, *vlpG* (locus tag EIH16_040; _070; _080; _0110; _0120; _07780; _07810; _7810) are classified as non-essential whereas genes for the variants *vlpB* and *vlpD* are shown to be essential (locus tag EIH16_050; _07790; _07820). However, the essentiality of the encoded Vlps cannot be defined with Tn-seq data since the reads could be mapped randomly among the *vlp* genes and multiple copies are encoded in the genome and because of respective elements and the highly homologous 90 bp of the prolipoprotein signal peptide among all Vlps [19]. Two predicted immunoglobulin A1 protease genes (*iga2* and *iga1*) are encoded in the genome of *M. hyorhinis,* similar to zinc metalloproteinase found in *Streptococcus*, which cleaves human immunoglobulin A1 in the hinge region. Gene *iga2* (locus tag EIH16_05330) is classified as non-essential whereas *iga1* (locus tag EIH16_02920) is classified as essential, which leaves the essentiality of the Iga system open. A similar mechanism for cleavage of IgG has been described in *Mycoplasma mycoides* subsp*. capri*, with a *Mycoplasma* Ig binding protein (MIB) and a *Mycoplasma* Ig protease (MIP) that have been functionally explored. We found orthologous genes in *M. hyorhinis* as well as in *M. hyopneumoniae* and thus not being classified as species-specific but non-essential in both species. The two homing HNH endonucleases (locus tag EIH16_02600; _03380), which enable DNA to move within and between genomes, are species-specific and non-essential for *M. hyorhinis.* Two predicted reductases were found to be unique and non-essential in *M. hyorhinis.* One is the peptide-methionine (S)-S-oxide reductase (locus tag EIH16_02570), which is involved in reactivating peptides by reducing oxidized methionine. However, the R form of this enzyme is also found in *M.* *hyopneumoniae* and classified as essential in both species. The other reductase (locus tag EIH16_0710) is a predicted MsnO8 family LLM class oxidoreductase described as a luciferase-like monooxygenase. The interconversion of l-aspartate to l-asparagine catalyzed by the aspartate-ammonia ligase (locus tag EIH16_02450) is also unique to *M. hyorhinis* and classified as non-essential. A Type I DNA restriction-modification system is found in *M. hyorhinis* and two components (S and R, locus tag EIH16_07470; _0200) out of three are unique to *M. hyorhinis*. The third subunit M is non-essential but not unique to *M. hyorhinis*. It is a methyltransferase and relates to a methyltransferase paeR7IM (locus tag EIH16_CDS 04270) found in *M. hyopneumoniae*. Moreover, a predicted DNA mismatch repair CDS (locus tag EIH16_01590) and a predicted integrase (locus tag EIH16_07720) are species-specific to *M. hyorhinis* and classified as non-essential. Amidohydrolases are a large family with various functions. One member of this family (locus tag EIH16_0760) is present in *M. hyorhinis* and is non-essential. An ATPase AAA (locus tag EIH16_01810) is classified as species-specific and non-essential to *M. hyorhinis* despite having various important cellular functions. Three CDS encoding domains involved in RNA processing, i.e. the RNA-binding S4 domain-containing protein (locus tag EIH16_03730), the S1 RNA-binding domain protein (locus tag EIH16_04810), and the class I SAM-dependent RNA methyltransferase (locus tag EIH16_05860) are also classified as species-specific and non-essential to *M. hyorhinis*. Finally, a unique SPFH/Band 7/PHB domain protein CDS (locus tag EIH16_04350) and a unique NERD domain-containing protein CDS (locus tag EIH16_01190) were shown to be non-essential.

## Discussion

The species-specific and non-essential CDS of *M. hyopneumoniae* strain F7.2C and of *M. hyorhinis* strain JF5820 were determined to narrowing down possible candidate genes that could confer to the different pathogenicity of these two phylogenetically related mycoplasmas found in the respiratory tract of pigs. Such genes could also be targets for developing attenuated live vaccines against both pathogens. For this purpose, the genome sequence of both strains was initially determined and transposon mutant libraries were generated. The Tn-seq method used in *Caulobacter crescentus* [13] was adapted to be applied in mycoplasmas. This included the design of optimized primers taking into account the low GC-content of mycoplasmas in the pentamers and in the wobble bases part of the semi-arbitrary primers. The advantage of the method compared to other Tn-seq techniques is that one only needs to perform PCRs on the culture of a pool of transposon mutants followed by high-throughput sequencing of amplification products. In other studies localization of transposon integrations were performed by either sequencing across the transposon junction into the genomic DNA for single mutants sequentially or by Tn-seq methods which include additional steps like DNA shearing, restriction or ligation [20, 21].

Our analysis resulted in a total of 101 species-specific non-essential CDS for *M. hyopneumoniae* and 96 species-specific non-essential CDS in *M.* *hyorhinis* (Additional files 1 and 2). Both gene pools included a high proportion of CDS being annotated as hypothetical proteins. These CDS are of interest for further investigation into their function and their contribution to the pathogenicity of *M.* *hyopneumoniae* and *M.* *hyorhinis*. The proportion of the gene pool with functionally annotated CDS allows for speculation about their role in pathogenicity and interaction with the host. In particular, entire pathways like the myo-inositol pathway in *M.* *hyopneumoniae* are non-essential and was already discussed as potentially related to virulence by comparing the genomes of porcine *Mycoplasma* species [4, 22]. This corroborates findings from in silico metabolic model analysis where this pathway was also suggested to contribute to pathogenicity [23, 24]. Since myo-inositol is freely available in the serum of pigs, it might be a suitable energy source for *M. hyopneumoniae* living in the highly vascularized lungs, thereby adapting to this niche. The catabolic pathway is composed of at least 10 CDS located on a transcriptional unit and among others leads to the production of acetyl coenzyme-A, an essential co-factor in many metabolic reactions. Furthermore, the genes *iolA, iolC* and *iolB* from the pathway locus have the highest number of transcript reads in the *M. hyopneumoniae* genome [25]. Despite these facts, we found the myo-inositol degradation pathway to be non-essential under ideal laboratory conditions, i.e. grown in rich medium and being devoid of competition for nutrients or energy sources. However, it might be an essential pathway in vivo for robust colonization of the lungs. Being essential for robust colonization in vivo and being non-essential for growth under ideal conditions could actually result in a candidate for a self-limiting live vaccine.

Production of cytotoxic H_2_O_2_ resulting from interconversion of glycerol by the glycerol-3-phosphate oxidase (GlpO) is considered a mycoplasma virulence mechanism [26]. GlpO was first described in *M.* *mycoides* subsp*. mycoides* SC [27]. Recently, it was experimentally shown that *M. hyopneumoniae* strains 7422 and 7448 are also able to produce H_2_O_2_ [24]. The orthologue genes of *glpO* are annotated as glycerol-3-phosphate dehydrogenase (*glpD*) in *M.* *hyopneumoniae* and *M. hyorhinis.* GlpD in these two species is assumed to be the corresponding oxidase GlpO in *M. mycoides* subsp*. mycoides* SC [24]. For *M. hyopneumoniae* strain F7.2C and of *M. hyorhinis* strain JF5820 the matching CDS was therefore annotated as *glpO* and it was shown to be non-essential for both. Further, an alternative pathway for glycerol uptake and interconversion to glycerol-6-phosphate was only present in *M. hyopneumoniae* and might allow for more efficient glycerol uptake and production of cytotoxic H_2_O_2_. This pathway involves the gene products of *glpF* and *glpK* with the latter being classified as non-essential. Given the fact that *glpO* is not found in the commensal *M. flocculare,* it is ranked high as a potential virulence gene in porcine mycoplasmas [4].

There are a total of eight serine proteases predicted in the *M. hyopneumoniae* strain F7.2C genome but only one subtilisin-like serine protease in *M. hyorhinis* strain JF5820. In addition, two serine proteases encoded in the genome of *M. hyopneumoniae* are unique and non-essential and thereby candidate genes to elucidate their difference in pathogenicity. The serine protease in *M. hyopneumoniae* strain F7.2C (locus tag EHI52_06040 corresponding to MHJ_0568 in strain J) was found to have a trypsin-like cleavage function and was shown to act on surface molecules in strain J [28]. Such cleavage alters surface topography; variation of surface molecules in turn also varies interaction with functionally and structurally diverse host molecules. Thereby we speculate that the serine proteases could play a role in immune evasion. Targeting genes involved in immune evasion for an attenuated live vaccine might thus allow the host to attain immunity against *M.* *hyopneumoniae* that will allow it to clear the pathogen when challenged and thereby avoid chronic infection.

The predicted sialic acid scavenging and degradation pathway is unique to *M. hyorhinis*. This pathway is composed of 12 CDS; two encoding sialidases, two encoding exo-alpha-sialidase, two encoding sialic acid transporter, two *nanA* genes, two *nanK* genes and two *nanE* genes. The sialidases are shedding off the extracellular localized sialic acids on eukaryotic cells [29] and have been shown to be associated with virulence of mycoplasmas. In *M. synoviae,* a poultry pathogen, the sialidase activity was experimentally proven with the highest level of sialidases activity observed in a highly pathogenic strain, which was associated with severe systemic disease in experimentally infected birds [30, 31]. A sialidase was also found in *M. alligatoris,* but not in *M. crocodyli,* which is seen as the reason for the attenuated virulence of the latter [32]. Consequently, we speculate that the sialidases in *M. hyorhinis* might pave the way for the spread from the respiratory tract to the joints of pigs. More downstream of the sialic acid scavenging and degradation pathway, sialic acid is degraded to *N*-acetyl-glucosamine-6-phosphate that can be delivered to the glycolysis pathway and might be seen as an alternative or extra energy source. We found that the CDS of the pathway, including the above mentioned sialidases are encoded in duplicates and with difference in their essentiality patterns, e.g. one copy is classified essential one is non-essential. Due to this redundancy, it is not possible to define the essentiality of those CDS in *M. hyorhinis*. Nevertheless, the redundancy of these CDS in an almost minimal bacterial cell, the orthologues in other mycoplasma pathogens associated with systemic infection and the sialic acid as a possible alternative energy source indicate an important role of the pathway in pathogenicity. Thus, to shut down the pathway might be an interesting way to produce a live attenuated vaccine. Further, the genome of *M. hyorhinis* encodes a glycosyl transferase, which is non-essential, and involved in covalently linking carbohydrates e.g. to proteins and lipids thereby contributing to capsule formation. Such a capsule can protect the pathogen against the host immune system thus being a virulence factor [33]. A mutant inhibited in capsule formation could be used as an attenuated live vaccine that might better expose the mycoplasmas surface antigens enabling the immune system to better act against *M. hyorhinis.*

The classification of non-essential genes remains putative, as insertions of transposons were not analyzed for functional disruption of the gene. Some genes might be misclassified due to redundancy or substitutional functionality of other genes. Moreover, we focused on the species-specific pool of non-essential genes, thereby excluding the common virulence associated genes that might be useful targets for attenuation in both species. Nevertheless, the analysis resulted in two sets of genes being the best candidates responsible for the difference in pathogenicity of the two species investigated. Functional individual gene analysis of these sets will certainly lead to a description of more virulence mechanisms. Determining differences in pathogenicity as described here is based on mostly putative functional annotation of genes, which might not reflect the true natural gene function in the two porcine mycoplasmas. However, the gene pools of unique non-essential genes also included well known virulence factors of mycoplasmas supporting that the defined gene pools harbour further potential virulence associated genes.

Identifying individual dispensable genes of porcine mycoplasmas is important for the development of novel vaccine approaches. The fact that a fully protective vaccine is not currently on the market for either species also indicates the difficulties in developing such a vaccine for chronic mycoplasma infections and improved approaches are needed. Currently used standard vaccines for *M. hyopneumoniae* are mostly bacterin-based vaccines. Thus, our study paves the way to develop a live vaccine by identifying and targeting non-essential genes to attenuate *M. hyopneumoniae* or *M. hyorhinis.*

In conclusion, we defined the unique and non-essential genes of *M. hyopneumoniae* and *M. hyorhinis* allowing the identification of possible targets for attenuation by gene disruption. Among these unique non-essential genes, we found already known virulence genes but also new candidate genes, including a high proportion of hypothetical protein CDS. Thus, investigating the function of these hypothetical protein CDS may form the basis to further elucidating the difference in pathogenicity of the two porcine *Mycoplasma* species.

## Additional files

**Additional file 1.** *Mycoplasma hyopneumoniae* strain F7.2C specific non-essential portion of coding sequences (CDS).

**Additional file 2.** *Mycoplasma hyorhinis* strain JF5820 specific non-essential portion of coding sequences (CDS).

## Data Availability

The datasets supporting the conclusions of this article are included within the article.
