# Supplementary material for: Tn-sequencing of Mycoplasma hyopneumoniae and Mycoplasma hyorhinis mutant libraries reveals non-essential genes of porcine mycoplasmas differing in pathogenicity
Source: Vet Res. 2019 Jul 19;50:55. doi: 10.1186/s13567-019-0674-7 (PMC6642558; doi:10.1186/s13567-019-0674-7)
Supplement: Supplementary file 1 — Additional file 1. Mycoplasma hyopneumoniae strain F7.2C specific non-essential portion of coding sequences (CDS). [file 13567_2019_674_MOESM1_ESM.docx]

**Additional file 1 *Mycoplasma hyopneumoniae* strain F7.2C specific non-essential portion of coding sequences (CDS)**.

| Locus tag  EHI52_ | CDS | Log fold change | edgeR_ *p*value | mean_  observed_  counts | mean_  pseudo  counts |
| --- | --- | --- | --- | --- | --- |
| 070 | hypothetical protein CDS | 0.023 | 0.614 | 58 | 59.468 |
| 0170 | hypothetical protein CDS | -0.302 | 1.000 | 49 | 63.084 |
| 0180 | hypothetical protein CDS | 0.964 | 0.327 | 178 | 94.612 |
| 0190 | hypothetical protein CDS | 2.441 | 0.046 | 315 | 60.412 |
| 0230 | hypothetical protein CDS | 1.634 | 0.168 | 180 | 60.356 |
| 0260 | hypothetical protein CDS | -1.356 | 0.926 | 28 | 74.894 |
| 0310 | hypothetical protein CDS | 3.777 | 0.001 | 587 | 44.552 |
| 0700 | hypothetical protein CDS | -3.017 | 0.720 | 3 | 26.062 |
| 0760 | hypothetical protein CDS | -3.408 | 0.689 | 3 | 34.154 |
| 01010 | hypothetical protein CDS | -0.702 | 1.000 | 82 | 138.958 |
| 01150 | hypothetical protein CDS | 1.343 | 0.147 | 774 | 318.002 |
| 01210 | IS1634-like element ISMhp1 family transposase CDS | 0.828 | 0.354 | 280 | 164.858 |
| 01230 | hypothetical protein CDS | -3.251 | 0.761 | 1 | 10.918 |
| 01270 | hypothetical protein CDS | 1.487 | 0.163 | 350 | 130.05 |
| 01330 | hypothetical protein CDS | -1.513 | 0.847 | 31 | 92.64 |
| 01340 | hypothetical protein CDS | -0.514 | 1.000 | 55 | 81.936 |
| 01640 | hypothetical protein CDS | 0.637 | 0.421 | 54 | 36.196 |
| 01850 | hypothetical protein CDS | -2.925 | 0.768 | 4 | 32.492 |
| 02370 | IS1634-like element ISMhp1 family transposase CDS | 0.056 | 0.691 | 172 | 172.322 |
| 02490 | hypothetical protein CDS | -0.937 | 0.990 | 60 | 120.328 |
| 02600 | methylmalonate-semialdehyde dehydrogenase *iolA* CDS | 1.416 | 0.167 | 389 | 151.786 |
| 02610 | 5-dehydro-2-deoxygluconokinase *iolC* CDS | 1.586 | 0.155 | 281 | 97.504 |
| 02620 | myo-inositol catabolism protein *iolB* CDS | -1.688 | 0.839 | 27 | 90.648 |
| 02640 | 3D-(3,5/4)-trihydroxycyclohexane-1,2-dione acylhydrolase (decyclizing) *iolD* CDS | 0.202 | 0.632 | 243 | 220.652 |
| 02650 | myo-inosose-2 dehydratase *iolE* CDS | 1.610 | 0.156 | 246 | 83.776 |
| 02680 | sugar ABC transporter substrate-binding protein CDS | -1.597 | 0.743 | 40 | 126.454 |
| 02690 | myo-inositol 2-dehydrogenase *iolX* CDS | -0.498 | 1.000 | 74 | 109.596 |
| 02710 | hypothetical protein CDS | -3.099 | 0.783 | 1 | 9.88 |
| 02720 | hypothetical protein CDS | -0.570 | 1.000 | 16 | 24.894 |
| 02820 | hypothetical protein CDS | 0.150 | 0.546 | 41 | 38.454 |
| 03050 | p116 (p102 paralog) CDS | -0.994 | 0.743 | 164 | 340.492 |
| 03250 | hypothetical protein CDS | -2.311 | 0.797 | 8 | 42.078 |
| 03260 | hypothetical protein CDS | 0.874 | 0.357 | 57 | 32.398 |
| 03280 | SAM-dependent DNA methyltransferase CDS | 0.610 | 0.439 | 313 | 213.908 |
| 03290 | hypothetical protein CDS | 0.944 | 0.333 | 157 | 84.8 |
| 03340 | hypothetical protein CDS | -2.851 | 0.789 | 3 | 23.376 |
| 03360 | hypothetical protein CDS | 0.972 | 0.322 | 190 | 101.104 |
| 03370 | hypothetical protein CDS | 2.897 | 0.013 | 565 | 79.014 |
| 03380 | hypothetical protein CDS | 1.711 | 0.165 | 64 | 20.16 |
| 03390 | hypothetical protein CDS | -2.349 | 0.368 | 42 | 223.586 |
| 03450 | hypothetical protein CDS | 1.174 | 0.162 | 1364 | 630.532 |
| 03500 | hypothetical protein CDS | 3.629 | 0.001 | 1195 | 100.442 |
| 03590 | IS1634-like element ISMhp1 family transposase CDS | 0.629 | 0.436 | 225 | 150.918 |
| 03780 | p102 paralog CDS | -1.452 | 0.567 | 99 | 282.672 |
| 03900 | glycerol kinase *glpK* CDS | -1.302 | 0.759 | 63 | 162.408 |
| 04050 | hypothetical protein CDS | -0.130 | 1.000 | 21 | 23.982 |
| 04080 | hypothetical protein CDS | 2.268 | 0.081 | 107 | 23 |
| 04090 | hypothetical protein CDS | 1.610 | 0.147 | 300 | 102.504 |
| 04110 | hypothetical protein CDS | -1.853 | 0.831 | 20 | 75.55 |
| 04120 | hypothetical protein CDS | -1.308 | 0.696 | 82 | 211.626 |
| 04170 | hypothetical protein CDS | -2.438 | 0.461 | 26 | 147.642 |
| 04180 | hypothetical protein CDS | 1.813 | 0.122 | 287 | 85.09 |
| 04190 | hypothetical protein CDS | 0.992 | 0.324 | 45 | 23.53 |
| 04200 | hypothetical protein CDS | -3.554 | 0.729 | 1 | 13.514 |
| 04210 | hypothetical protein CDS | 2.016 | 0.107 | 178 | 45.71 |
| 04240 | hypothetical protein CDS | -3.125 | 0.595 | 7 | 64.424 |
| 04250 | serine protease CDS | -1.704 | 0.552 | 64 | 217.716 |
| 04260 | hypothetical protein CDS | -0.867 | 0.934 | 89 | 169.518 |
| 04270 | hypothetical protein CDS | -1.095 | 0.976 | 40 | 89.31 |
| 04280 | hypothetical protein CDS | -1.765 | 0.781 | 25 | 88.482 |
| 04290 | hypothetical protein CDS | -2.806 | 0.570 | 12 | 88.214 |
| 04330 | hypothetical protein CDS | -0.646 | 1.000 | 11 | 18.066 |
| 04340 | serine protease CDS | -0.628 | 1.000 | 32 | 51.598 |
| 04350 | hypothetical protein CDS | -1.118 | 0.829 | 74 | 167.888 |
| 04360 | IS1634-like element ISMhp1 family transposase CDS | 0.532 | 0.475 | 236 | 169.932 |
| 04480 | hypothetical protein CDS | -1.253 | 1.000 | 22 | 55.12 |
| 04580 | hypothetical protein CDS | 0.916 | 0.341 | 176 | 97.07 |
| 04590 | hypothetical protein CDS | 2.819 | 0.031 | 79 | 11.566 |
| 04670 | hypothetical protein CDS | 0.884 | 0.339 | 258 | 145.978 |
| 04960 | hypothetical protein CDS | -0.318 | 1.000 | 71 | 92.252 |
| 04970 | hypothetical protein CDS | 1.658 | 0.092 | 650 | 214.69 |
| 05180 | hypothetical protein CDS | 2.022 | 0.050 | 650 | 167.028 |
| 05200 | hypothetical protein CDS | 1.141 | 0.279 | 132 | 62.076 |
| 05310 | MFS transporter CDS | 0.763 | 0.385 | 210 | 129.018 |
| 05320 | hypothetical protein CDS | -2.314 | 0.448 | 36 | 187.084 |
| 05560 | hypothetical protein CDS | -3.646 | 0.712 | 1 | 14.41 |
| 05670 | hypothetical protein CDS | 0.542 | 0.452 | 82 | 59.068 |
| 05800 | hypothetical protein CDS | -3.193 | 0.705 | 3 | 29.406 |
| 05810 | hypothetical protein CDS | 2.044 | 0.055 | 527 | 132.976 |
| 05900 | PTS galacitol transporter subunit IIB CDS | 1.960 | 0.120 | 111 | 29.596 |
| 05920 | hypothetical protein CDS | -0.156 | 1.000 | 29 | 33.574 |
| 05940 | hypothetical protein CDS | -2.653 | 0.652 | 10 | 66.208 |
| 05970 | hypothetical protein CDS | -1.391 | 0.670 | 72 | 196.598 |
| 05980 | hypothetical protein CDS | -3.458 | 0.236 | 16 | 184.886 |
| 05990 | hypothetical protein CDS | 2.152 | 0.041 | 619 | 144.994 |
| 06000 | hypothetical protein CDS | -3.245 | 0.252 | 18 | 178.994 |
| 06010 | hypothetical protein CDS | 1.891 | 0.070 | 533 | 149.482 |
| 06020 | hypothetical protein CDS | -1.024 | 0.834 | 90 | 191.212 |
| 06030 | hypothetical protein CDS | 2.477 | 0.016 | 806 | 150.596 |
| 06040 | serine protease CDS | -1.546 | 0.914 | 21 | 64.45 |
| 06050 | IS1634-like element ISMhp1 family transposase CDS | -0.381 | 1.000 | 122 | 165.74 |
| 06070 | N-acetylglucosamine-6-phosphate deacetylase *nagA* CDS | 0.762 | 0.388 | 190 | 117.16 |
| 06340 | hypothetical protein CDS | -2.744 | 0.415 | 20 | 140.894 |
| 06360 | hypothetical protein CDS | -0.769 | 0.937 | 108 | 192.246 |
| 06370 | hypothetical protein CDS | -0.039 | 1.000 | 139 | 149.158 |
| 06380 | hypothetical protein CDS | 0.741 | 0.382 | 302 | 188.14 |
| 06490 | *crr* CDS | -1.033 | 1.000 | 30 | 63.868 |
| 06970 | IS1634-like element ISMhp1 family transposase CDS | 0.623 | 0.436 | 286 | 193.754 |
| 06980 | p102 paralog CDS | -1.212 | 0.613 | 162 | 391.258 |
| 07040 | hypothetical protein CDS | -3.320 | 0.747 | 1 | 11.494 |
| 07120 | *ydiS* CDS | -0.030 | 1.000 | 257 | 274.112 |
