# Supplementary material for: Tn-sequencing of Mycoplasma hyopneumoniae and Mycoplasma hyorhinis mutant libraries reveals non-essential genes of porcine mycoplasmas differing in pathogenicity
Source: Vet Res. 2019 Jul 19;50:55. doi: 10.1186/s13567-019-0674-7 (PMC6642558; doi:10.1186/s13567-019-0674-7)
Supplement: Supplementary file 2 — Additional file 2. Mycoplasma hyorhinis strain JF5820 specific non-essential portion of coding sequences (CDS). [file 13567_2019_674_MOESM2_ESM.docx]

**Additional file 2 *Mycoplasma hyorhinis* strain JF5820 specific non-essential portion of coding sequences (CDS).**

| Locus tag  EIH16_ | CDS | Log fold change | edgeR_ *p*value | mean_  observed_  counts | mean_  pseudo  counts |
| --- | --- | --- | --- | --- | --- |
| 040 | lipoprotein *vlpC_1* CDS | 3.107 | 0.003 | 812 | 87.618 |
| 070 | lipoprotein *vlpA_1* CDS | 2.917 | 0.004 | 926 | 114.256 |
| 080 | lipoprotein *vlpE_1* CDS | -2.914 | 0.447 | 13 | 92.294 |
| 0110 | lipoprotein *vlpF* CDS | 0.024 | 0.694 | 130 | 119.444 |
| 0120 | lipoprotein *vlpE_2* CDS | -0.509 | 1.000 | 67 | 88.85 |
| 0170 | hypothetical protein CDS | -3.420 | 0.743 | 1 | 11.226 |
| 0180 | hypothetical protein CDS | -2.977 | 0.238 | 31 | 228.436 |
| 0200 | type I restriction endonuclease subunit R CDS | 0.427 | 0.524 | 399 | 276.702 |
| 0710 | MsnO8 family LLM class oxidoreductase CDS | -0.987 | 0.990 | 42 | 77.716 |
| 0760 | amidohydrolase CDS | -2.395 | 0.456 | 26 | 128.172 |
| 0870 | glycosyl transferase CDS | -2.520 | 0.583 | 14 | 75.698 |
| 01040 | hypothetical protein CDS | -3.851 | 0.674 | 1 | 15.17 |
| 01110 | hypothetical protein CDS | -3.389 | 0.362 | 9 | 89.292 |
| 01190 | NERD domain-containing protein CDS | -3.531 | 0.511 | 5 | 55.118 |
| 01290 | hypothetical protein CDS | -3.514 | 0.716 | 1 | 11.994 |
| 01540 | acid phosphatase CDS | -2.268 | 0.536 | 24 | 108.196 |
| 01590 | DNA mismatch repair CDS | -2.070 | 0.542 | 32 | 125.882 |
| 01600 | restriction endonuclease subunit S CDS | -2.446 | 0.539 | 19 | 97.182 |
| 01620 | hypothetical protein CDS | -3.255 | 0.212 | 22 | 196.782 |
| 01630 | hypothetical protein CDS | -2.715 | 0.715 | 7 | 43.698 |
| 01710 | hypothetical protein CDS | -3.422 | 0.199 | 20 | 200.616 |
| 01810 | ATPase AAA CDS | -1.680 | 0.553 | 63 | 188.896 |
| 01870 | sucrase-isomaltase CDS | 0.417 | 0.525 | 179 | 124.806 |
| 01990 | hypothetical protein CDS | -2.141 | 0.725 | 15 | 62.212 |
| 02000 | hypothetical protein CDS | -2.602 | 0.761 | 7 | 40.134 |
| 02150 | hypothetical protein CDS | -0.474 | 1.000 | 15 | 19.524 |
| 02300 | hypothetical protein CDS | 2.957 | 0.021 | 189 | 22.68 |
| 02350 | hypothetical protein CDS | -0.636 | 1.000 | 24 | 34.84 |
| 02420 | hypothetical protein CDS | -1.579 | 0.981 | 11 | 30.944 |
| 02450 | aspartate--ammonia ligase CDS | 1.285 | 0.226 | 220 | 84.152 |
| 02570 | peptide-methionine (S)-S-oxide reductase CDS | -3.697 | 0.571 | 3 | 37.818 |
| 02600 | HNH endonuclease CDS | -3.006 | 0.410 | 13 | 98.35 |
| 02860 | hypothetical protein CDS | -3.693 | 0.516 | 4 | 49.756 |
| 02930 | hypothetical protein CDS | -0.263 | 1.000 | 27 | 30.218 |
| 03020 | hypothetical protein CDS | 3.547 | 0.003 | 370 | 29.266 |
| 03060 | hypothetical protein CDS | -3.872 | 0.658 | 1 | 15.36 |
| 03270 | hypothetical protein CDS | -0.145 | 1.000 | 60 | 61.744 |
| 03380 | HNH endonuclease CDS | 1.502 | 0.195 | 137 | 45.03 |
| 03660 | hypothetical protein CDS | 1.950 | 0.122 | 64 | 15.274 |
| 03680 | hypothetical protein CDS | -2.710 | 0.711 | 7 | 43.368 |
| 03730 | RNA-binding S4 domain-containing protein CDS | -2.217 | 0.907 | 4 | 17.758 |
| 03800 | hypothetical protein CDS | 0.631 | 0.428 | 333 | 200.348 |
| 03810 | hypothetical protein CDS | -3.152 | 0.451 | 9 | 75.708 |
| 03820 | hypothetical protein CDS | -3.797 | 0.555 | 3 | 40.512 |
| 03860 | hypothetical protein CDS | -0.755 | 1.000 | 11 | 17.324 |
| 03890 | hypothetical protein CDS | -2.912 | 0.342 | 18 | 127.096 |
| 03900 | hypothetical protein CDS | -3.469 | 0.739 | 1 | 11.592 |
| 03910 | hypothetical protein CDS | -0.109 | 1.000 | 60 | 60.26 |
| 03930 | hypothetical protein CDS | -2.297 | 0.538 | 23 | 106.038 |
| 04020 | hypothetical protein CDS | -1.691 | 0.652 | 39 | 117.806 |
| 04050 | hypothetical protein CDS | -3.641 | 0.598 | 3 | 36.146 |
| 04090 | hypothetical protein CDS | 1.932 | 0.074 | 428 | 104.406 |
| 04230 | hypothetical protein CDS | -1.937 | 0.894 | 8 | 29.004 |
| 04340 | hypothetical protein CDS | 1.046 | 0.307 | 95 | 42.956 |
| 04350 | SPFH/Band 7/PHB domain protein CDS | 3.402 | 0.002 | 803 | 70.638 |
| 04600 | hypothetical protein CDS | 0.853 | 0.374 | 23 | 11.854 |
| 04640 | hypothetical protein CDS | -2.255 | 0.892 | 5 | 22.832 |
| 04650 | hypothetical protein CDS | 0.131 | 0.566 | 30 | 25.508 |
| 04810 | S1 RNA-binding domain protein CDS | -1.831 | 0.900 | 11 | 36.842 |
| 04860 | hypothetical protein CDS | -1.968 | 0.493 | 45 | 164.864 |
| 04930 | hypothetical protein CDS | -2.002 | 0.778 | 15 | 56.498 |
| 04940 | N-acetylmannosamine-6-phosphate 2-epimerase *nanE_1* CDS | -1.129 | 1.000 | 11 | 22.664 |
| 04960 | hypothetical protein CDS | -3.301 | 0.766 | 1 | 10.294 |
| 04990 | sialic acid transporter_2 CDS | 1.212 | 0.265 | 106 | 42.606 |
| 05000 | hypothetical protein CDS | -1.606 | 1.000 | 4 | 11.64 |
| 05010 | N-acetylneuraminate lyase *nanA_1* CDS | 0.971 | 0.335 | 40 | 18.96 |
| 05020 | N-acetylneuraminate lyase *nanA_2* CDS | -2.532 | 0.848 | 3 | 16.794 |
| 05040 | N-acetylmannosamine kinase *nanK_2* CDS | -2.511 | 0.801 | 6 | 32.648 |
| 05140 | hypothetical protein CDS | -3.408 | 0.615 | 4 | 40.768 |
| 05270 | exo-alpha-sialidase_2 CDS | -3.372 | 0.527 | 6 | 59.048 |
| 05280 | hypothetical protein CDS | -1.285 | 1.000 | 13 | 29.722 |
| 05330 | *iga2* CDS | -0.187 | 1.000 | 197 | 209.468 |
| 05340 | alpha-amylase CDS | 0.108 | 0.684 | 232 | 200.93 |
| 05360 | hypothetical protein CDS | -3.648 | 0.700 | 1 | 13.228 |
| 05440 | hypothetical protein CDS | -3.077 | 0.804 | 1 | 8.792 |
| 05750 | hypothetical protein CDS | -0.184 | 1.000 | 184 | 195.368 |
| 05850 | hypothetical protein CDS | 4.383 | 0.000 | 587 | 26.08 |
| 05860 | class I SAM-dependent RNA methyltransferase CDS | -3.824 | 0.318 | 7 | 94.45 |
| 06190 | hypothetical protein CDS | -1.316 | 0.819 | 44 | 102.728 |
| 06220 | hypothetical protein CDS | 0.143 | 0.613 | 96 | 80.996 |
| 06450 | hypothetical protein CDS | -2.079 | 0.748 | 15 | 59.602 |
| 06580 | hypothetical protein CDS | -3.032 | 0.750 | 3 | 23.89 |
| 06610 | hypothetical protein CDS | -0.765 | 1.000 | 27 | 43.078 |
| 06640 | hypothetical protein CDS | -3.535 | 0.718 | 1 | 12.252 |
| 06820 | hypothetical protein CDS | -3.336 | 0.490 | 7 | 67.202 |
| 06950 | serine/threonine-protein phosphatase CDS | 0.359 | 0.525 | 86 | 62.392 |
| 07470 | restriction endonuclease subunit S CDS | -2.871 | 0.422 | 15 | 103.236 |
| 07480 | hypothetical protein CDS | -3.394 | 0.740 | 1 | 10.988 |
| 07520 | hypothetical protein CDS | -0.655 | 1.000 | 11 | 16.2 |
| 07530 | hypothetical protein CDS | 0.945 | 0.335 | 92 | 44.466 |
| 07550 | hypothetical protein CDS | -1.373 | 1.000 | 7 | 17.164 |
| 07560 | hypothetical protein CDS | -3.060 | 0.751 | 3 | 24.338 |
| 07720 | site-specific integrase CDS | -3.512 | 0.376 | 8 | 86.36 |
| 07780 | lipoprotein *VlpC_2* CDS | 3.094 | 0.003 | 777 | 84.748 |
| 07810 | lipoprotein *VlpA_2* CDS | 2.947 | 0.004 | 895 | 108.574 |
| 07820 | lipoprotein *VlpG* CDS | -2.452 | 0.536 | 19 | 97.514 |
